# Anti-HIV-1 reverse transcriptase property of some edible mushrooms in Asia

**DOI:** 10.1016/j.sjbs.2021.02.012

**Published:** 2021-02-16

**Authors:** Khuanjarat Choengpanya, Siriluk Ratanabunyong, Supaphorn Seetaha, Lueacha Tabtimmai, Kiattawee Choowongkomon

**Affiliations:** a Program in Applied Biology, Maejo University Phrae Campus, Phrae 54140, Thailand; b Department of Biochemistry, Faculty of Science, Kasetsart University, Bangkok 10900, Thailand; c Interdisciplinary Graduate Program in Bioscience, Faculty of Science, Kasetsart University, Bangkok 10900, Thailand; d Department of Companion Animals Clinical Sciences, Faculty of Faculty of Veterinary Medicine, Kasetsart University, Bangkok 10900, Thailand; e Center for Advanced Studies for Agriculture and Food, Kasetsart University Institute for Advanced Studies, Kasetsart University, Bangkok 10900, Thailand

**Keywords:** HIV-1 reverse transcriptase, Anti-HIV-1 agents, Mushrooms, Crude extracts

## Abstract

Human immunodeficiency virus (HIV) causes acquired immunodeficiency syndrome (AIDS), which is a serious health threat worldwide. One of its core enzymes, reverse transcriptase (RT), is a target for HIV inhibition. A number of bioactive compounds have been successfully used for HIV treatment. However, HIV rapidly mutates, and long-term treatment can cause drug-resistant strains. Therefore, new inhibitors are required to overcome this problem. In this study, the aqueous, ethanolic and hexane crude extracts of 19 edible and medicinal mushrooms, which are widely grown and available commercially in Thailand, were screened against HIV-1 RT. The results showed that the water extracts of *A. blazei* and *I. obliquus*, the ethanol extracts of *I. obliquus* and *P. igniarius* and the hexane extract of *I. obliquus* exhibited strong anti-HIV-1 RT activity with IC_50_ values of 1.92 ± 0.15, 4.39 ± 0.79, 6.17 ± 0.76 and 7.75 ± 246 µg/ml, respectively. These mushrooms have the potential for HIV treatment, and further study on identification of the bioactive compounds against HIV-1 RT should be performed.

## 1 Introduction

The human Immunodeficiency virus (HIV) is one of the most potent infectious viruses. It causes acquired immunodeficiency syndrome or AIDS, which has spread worldwide, especially in Africa and Asia. Nowadays, approximately 36.9 million people are living with HIV/AIDS, and there are about 5,000 newly infected people daily, making HIV a serious health threat (​Global Statistics, 2019).

There are various commercial drugs used for HIV-1 treatment. These drugs can be divided into 6 types according to their targets such as reverse transcriptase (RT), protease, CCR5 receptor and integrase. As RT is a crucial enzyme for viral propagation in the host cell, it is targeted by a large number of inhibitors. There are 2 types of HIV-1 RT inhibitors, nucleotide/nucleoside RT inhibitors (NRTIs) and non-nucleoside RT inhibitors (NNRTIs). NRTIs, which resemble the nucleotide substrates of HIV-1 RT, target the catalytic site of the enzyme, causing inhibition of its activity. However, NRTIs can also inhibit cellular polymerase enzymes. Thus, NRTIs have highly toxic side effects. Unlike the NRTIs, NNRTIs target the allosteric site of HIV-1 RT, called non-nucleoside inhibitor binding pocket or NNIBP, which is approximately 10 Å away from the catalytic site of HIV-1 RT (Sluis-Cremer and Tachedjian, 2008). NNRTIs are structurally diverse, highly selective and less cytotoxic. Both types of inhibitors are used together to treat HIV-1 infected patients, and long-term treatment with these drugs can cause drug-resistant variants. The first-generation NNRTIs, such as nevirapine, possess a rigid butterfly-like structure, consisting of a central diazepine domain attached to two heterocyclic hydrophobic wing domains. The central domain hydrogen bonds with K101, K103, and P236 residues, while the wing domains establish hydrophobic interactions with the amino acids Y181, Y188, W229, F227, V106, P236, L100, L232, and Y318 of NNIBP (Smerdon et al., 1994). The most frequent single-point mutations, such as K103N, Y181C and Y188L, cause alteration of hydrogen bonding and hydrophobic interactions between nevirapine and the NNIBP. Moreover, mutations also cause alteration in the shape of NNIBP (Smerdon et al., 1994). Second and third generation NNRTIs have been developed, which possess more flexible horseshoe-like structures. The flexibility allows these NNRTIs to tolerate these single-point mutations (Wang et al., 2020). However, emergence of double mutations caused resistance to the second and the third generation NNRTIs (Battini and Bollini, 2019). Therefore, new inhibitors with better structural adaptability are required to overcome this problem.

Almost 144,000 fungal species have been reported to date, which makes fungi a promising source of new inhibitors against HIV-1 RT (Niskanen et al., 2018). Many researchers have reported various pharmacological potentials of mushroom extracts such as antimicrobial, antitumor, anti-inflammatory, antiviral and immunomodulating activities (Lindequist et al., 2005; Duru and Cayan, 2015). For example, triterpenes from the mushroom *Ganoderma lucidum* have been shown to inhibit HIV-1 protease and HIV-1 induced cytopathic effects in MT-4 cells (El-Mekkawy et al., 1998). Other anti-HIV-1 substances such as ganoderiol F, ganodermanontriol and ganoderic acid B from *G. lucidum*, lentinan from *L. edodes*, velutin from *F. velutipes*, laccase from *Volvariella volvacea*, and *p*-hydroxybenzoic (PHBA), *p*-coumaric and cinnamic acids from *Pleurotus sajor-caju* also showed anti-HIV-1 protease and reverse transcriptase activities (Lindequist et al., 2005; Duru and Cayan , 2015). Therefore, mushrooms are excellent sources of promising pharmaceutical agents. Nineteen edible and medicinal mushrooms, which are widely distributed in Thailand, were used in this study. The inhibition assays and determination of IC_50_ were performed by the fluorescence method. The IC_50_ indicated that these mushroom extracts can be potential anti-HIV-1 RT agents.

## 2 Materials and methods

### 2.1 Chemicals

All chemicals used were of analytical grade except ethanol and hexane, which were of reagent grade. EnzChek® reverse transcriptase assay kit was purchased from Molecular Probes (USA). Nevirapine (NVP) was from Government Pharmaceutical Organization Thailand (GPO).

### 2.2 Mushroom samples and preparation of mushroom crude extracts

**Fig. 1:**
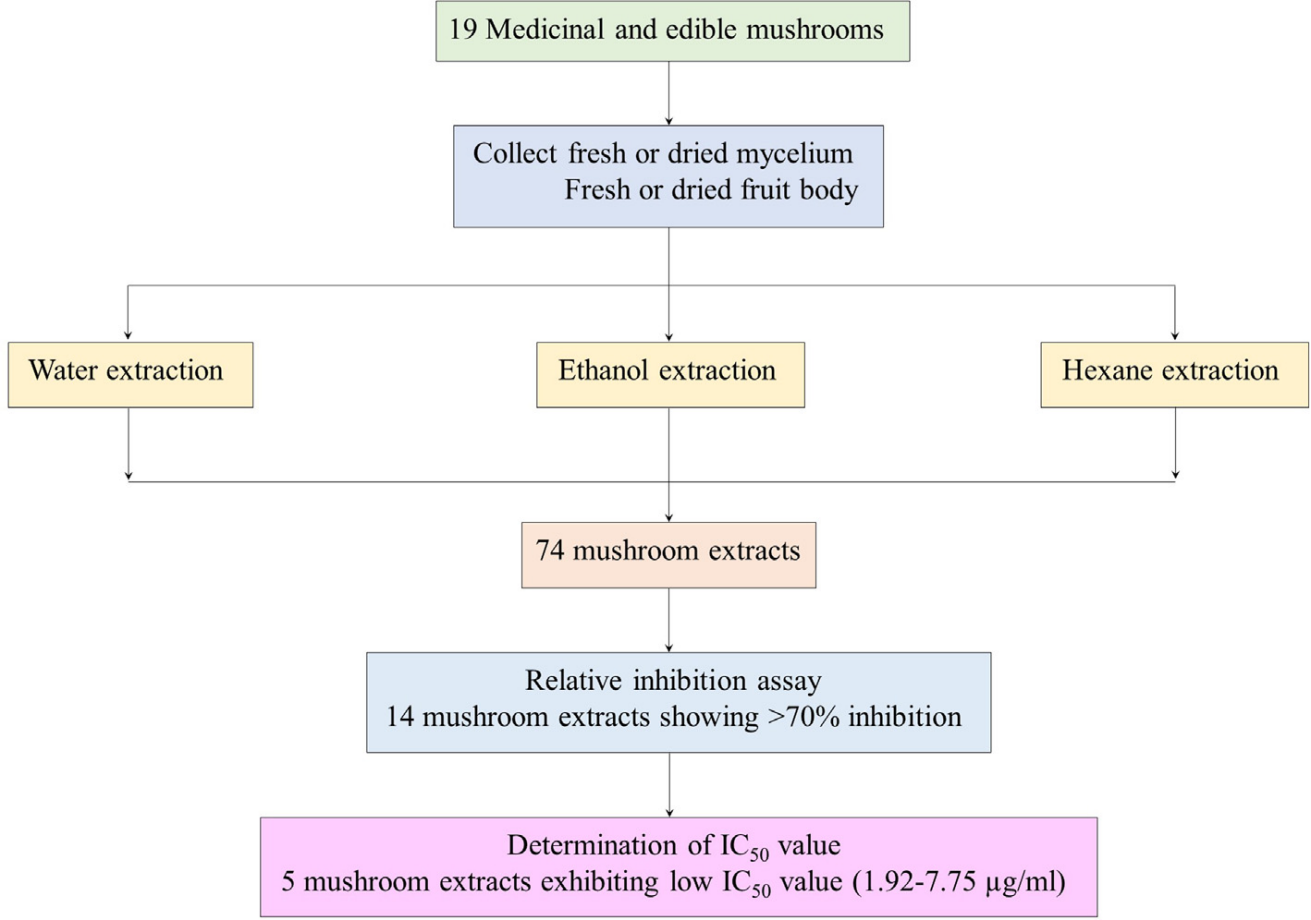
Diagram represents workflow of this study.

The taxonomic identification of the 19 edible and medicinal mushrooms used in this study was performed by comparing the external morphological characteristic such as shape, color and surface texture of pileus, gills and stalk with monographs printed in Thai language (Chandrasrikul et al., 2008, Sangwanit et al., 2013). Some mushrooms, namely *Agaricus subrufescens*, *Cordyceps militaris*, *Ganoderma lingzhi* and *Hericium erinaceus* which could not be identified by monographs, were identified by DNA sequencing. These mushrooms were *A. subrufescens*, *C. militaris*, *Dictyophora indusiata*, *Flammulina velutipes*, *G. lingzhi*, *H. erinaceus*, *Hypsizygus marmoreus*, *Inonotus obliquus*, *Lentinula edodes*, *Lentinus aquarrosulus*, *Lentinus* TAFRS007, *Lentinus* TAFRS011, *Lentinus* TAFRS014, *Morchella esculenta*, *Phellinus igniarius*, *Pleurotus eryngii*, *Pleurotus sajor-caju*, *Tremella fuciformis* and *Volvariella volvacea.* They were collected from local markets in Thailand, and were cut into small pieces, ground and lyophilized for 2 days. After that, the dried small pieces of mushrooms were ground into powder, and divided into 3 parts which were then extracted with each solvent, which included distilled water, 99.5% ethanol and hexane (Fig. 1). The temperature used in the extraction process followed the studies of Faccin et al., 2007, Yim et al., 2012. Extraction using water as the solvent was performed at 25 °C in a shaker at 180 rpm overnight (Faccin et al., 2007). Yim et al. (2012) has reported that the optimal extraction temperatures for *P. porrigens* were 32.0–46.6 °C, so the temperature of 37 °C was chosen for extraction with ethanol and hexane as solvents overnight in a shaker at the same rpm. After incubation, all crude extracts were filtered through Whatman no. 1 filter papers and centrifuged at 7,000 rpm, 4 °C for 15 min to remove precipitate. The ethanol and hexane solvents were removed by using a hot-air oven at 80 °C, and the DW was removed by lyophilization. The mushroom powder was re-dissolved in 100% DMSO to a final concentration of 100 mg/ml. Seventy four extracts were obtained from the 19 mushroom species examined (Table 1). The crude extracts were kept at −20 °C until used.

**Table 1:** Selected mushrooms, parts used, solvents used and codes. Fresh and dried fruiting bodies and mycelial powder were derived from food and food supplements. Therefore, these parts were selected and used in this study.

**Scientific name**	**Part used**	**Solvent used**	**Code**
*Agaricus subrufescens*	Fresh mycelium	Distilled water	*A. subrufescens* Mf-DW
	Dried mycelium	Distilled water	*A. subrufescens*Md-DW
	Dried fruiting body	Distilled water	*A. subrufescens*D-DW
	Fresh mycelium	Ethanol	*A. subrufescens*Mf-EtOH
	Dried mycelium	Ethanol	*A. subrufescens*Md-EtOH
	Dried fruiting body	Ethanol	*A. subrufescens*D-EtOH
	Dried mycelium	Hexane	*A. subrufescens*Md-Hex
	Dried fruiting body	Hexane	*A. subrufescens*D-Hex
*Cordyceps militaris*	Fresh fruiting body	Distilled water	*C. militaris*-DW
	Fresh fruiting body	Ethanol	*C. militaris*-EtOH
	Fresh fruiting body	Hexane	*C. militaris*-Hex
*Dictyophora indusiata*	Fresh mycelium	Distilled water	*D. indusiata*Mf-DW
	Dried mycelium	Distilled water	*D. indusiata*Md-DW
	Fresh fruiting body	Distilled water	*D. indusiata*F-DW
	Dried fruiting body	Distilled water	*D. indusiata*D-DW
	Fresh mycelium	Ethanol	*D. indusiata*Mf-EtOH
	Dried mycelium	Ethanol	*D. indusiata*Md-EtOH
	Fresh fruiting body	Ethanol	*D. indusiata*F-EtOH
	Dried fruiting body	Ethanol	*D. indusiata*D-EtOH
	Fresh mycelium	Hexane	*D. indusiata*Mf-Hex
	Dried mycelium	Hexane	*D. indusiata*Md-Hex
	Fresh fruiting body	Hexane	*D. indusiata*F-Hex
	Dried fruiting body	Hexane	*D. indusiata*D-Hex
*Flammulina velutipes*	Fresh fruiting body	Distilled water	*F. velutipes*-DW
	Fresh fruiting body	Ethanol	*F. velutipes*-EtOH
	Fresh fruiting body	Hexane	*F. velutipes*-Hex
*Ganoderma lingzhi*	Fresh fruiting body	Distilled water	*G. lingzhi-*DW
	Fresh fruiting body	Ethanol	*G. lingzhi-*EtOH
	Fresh fruiting body	Hexane	*G. lingzhi-*Hex
*Hericium erinaceus*	Fresh fruiting body	Distilled water	*H. erinaceus*-DW
*Hypsizygus marmoreus*	Fresh fruiting body	Distilled water	*H. marmoreus*-DW
	Fresh fruiting body	Ethanol	*H. marmoreus*-EtOH
	Fresh fruiting body	Hexane	*H. marmoreus*-Hex
*Inonotus obliquus*	Fresh mycelium	Distilled water	*I. obliquus*Mf-DW
	Dried mycelium	Distilled water	*I. obliquus*Md-DW
	Dried fruiting body	Distilled water	*I. obliquus*D-DW
	Fresh mycelium	Ethanol	*I. obliquus*Mf-EtOH
	Dried mycelium	Ethanol	*I. obliquus*Md-EtOH
	Dried fruiting body	Ethanol	*I. obliquus*D-EtOH
	Fresh mycelium	Hexane	*I. obliquus*Mf-Hex
	Dried mycelium	Hexane	*I. obliquus*Md-Hex
	Dried fruiting body	Hexane	*I. obliquus*D-Hex
*Lentinula edodes*	Fresh fruiting body	Distilled water	*L. edodes*-DW
	Fresh fruiting body	Ethanol	*L. edodes*-EtOH
	Fresh fruiting body	Hexane	*L. edodes*-Hex
*Lentinus squarrosulus*	Fresh fruiting body	Distilled water	*L. squarrosulus*-DW
	Fresh fruiting body	Ethanol	*L. squarrosulus*-EtOH
*Lentinus* TAFRS007	Dried mycelium	Distilled water	TAFRS007-DW
	Dried mycelium	Ethanol	TAFRS007-EtOH
	Dried mycelium	Hexane	TAFRS007-Hex
*Lentinus* TAFRS011	Dried mycelium	Distilled water	TAFRS011-DW
	Dried mycelium	Ethanol	TAFRS011-EtOH
	Dried mycelium	Hexane	TAFRS011-Hex
*Lentinus* TAFRS014	Dried mycelium	Distilled water	TAFRS014-DW
	Dried mycelium	Ethanol	TAFRS014-EtOH
	Dried mycelium	Hexane	TAFRS014-Hex
*Morchella esculenta*	Fresh fruiting body	Distilled water	*M. esculenta*-DW
	Fresh fruiting body	Ethanol	*M. esculenta*-EtOH
	Fresh fruiting body	Hexane	*M. esculenta*-Hex
*Phellinus igniarius*	Fresh fruiting body	Distilled water	*P. igniarius*-DW
	Fresh fruiting body	Ethanol	*P. igniarius*-EtOH
	Fresh fruiting body	Hexane	*P. igniarius*-Hex
*Pleurotus eryngii*	Fresh fruiting body	Distilled water	*P. eryngii*-DW
	Fresh fruiting body	Ethanol	*P. eryngii*-EtOH
	Fresh fruiting body	Hexane	*P. eryngii*-Hex
*Pleurotus sajor-caju*	Fresh fruiting body	Distilled water	*P. sajor-caju*-DW
	Fresh fruiting body	Ethanol	*P. sajor-caju*-EtOH
	Fresh fruiting body	Hexane	*P. sajor-caju*-Hex
*Tremella fuciformis*	Fresh fruiting body	Distilled water	*T. fuciformis*-DW
	Fresh fruiting body	Ethanol	*T. fuciformis*-EtOH
	Fresh fruiting body	Hexane	*T. fuciformis*-Hex
*Volvariella volvacea*	Fresh fruiting body	Distilled water	*V. volvacea*-DW
	Fresh fruiting body	Ethanol	*V. volvacea*-EtOH
	Fresh fruiting body	Hexane	*V. volvacea*-Hex

### 2.3 HIV-1 reverse transcriptase relative inhibition assay

EnzChek® reverse transcriptase assay kit (Molecular Probes, USA) was used in this study. The relative inhibition assay was performed by using the fluorescence method according to Silprasit et al. (2011). Briefly, 2 µl of 10 mg/ml of the mushroom extracts were added into each well of a 384-well plate containing 13 µl HIV-1 RT reaction buffer (50 mM TE pH 7.6, 2 mM DTT, 20% glycerol). Then, 5 µl of 50 nM purified recombinant wild-type HIV-1 RT was added into each well. The reaction was started by adding 5 µl of the 1:400 primer/template substrate. The reaction was incubated at 37 °C for 30 min, and was stopped by adding 5 µl 0.2 M EDTA. Next, 40 µl of 1:700 Picogreen was added in the stopped reaction. The reaction was then incubated in the dark for 5 min, and the fluorescence was measured at an excitation wavelength of 485 nm and an emission wavelength of 535 nm (RTsample). 100% DMSO and 0.2 M EDTA were used instead of mushroom extract to serve as positive (RTpositive) and background (RTbackground) reactions, respectively. The HIV-1 RT inhibitor nevirapine (NVP) was added instead of mushroom extract for the positive control reaction. Three independent experiments were performed. The percent relative inhibition was calculated following the methods of Silprasit et al. (2011), which was calculated from [(RTpositive-RTbackground)-(RTsample-RTbackground)]x100/(RTpositive-RTbackground). The extracts showing high relative inhibition (>70% relative inhibition) were selected for the IC_50_ assay.

### 2.4 Determination of IC_50_

Determination of IC_50_ value was performed according to the methods of Silprasit et al. (2011), which have been used in a number of researches (Bahare et al., 2015, Seetaha et al., 2020). Briefly, the 6–14 concentrations of two-fold serial dilution of the concentrated mushroom crude extract were prepared, and were then used as inhibitors in the assay. Two µl of each two-fold diluted mushroom extract was mixed with 13 µl HIV-1 RT reaction buffer in a 384-well plate. Then, 5 µl of 50 nM purified recombinant wild-type HIV-1 RT were added into each well. The reaction was started by adding 5 µl of 1:400 primer/template. The reaction was incubated, stopped and the fluorescence intensity was measured as previously described. The fluorescence intensity of each mushroom concentration was transformed into percent relative inhibition following the equation previously described. Six to fourteen values of percent relative inhibition for each mushroom were fitted with the nonlinear regression dose response curve [log(inhibitor) vs. response – variable slope (four parameters) equation], which was generated by using the IC_50_ function in GraphPad Prism program (GraphPad Software Inc., San Diego, CA, USA).

## 3 Results

### 3.1 Mushroom crude extracts

The 19 mushrooms, namely *A subrufescens*, *C. militaris*, *D. indusiata*, *F. velutipes*, *G. lingzhi*, *H. erinaceus*, *H. marmoreus*, *I. obliquus*, *L. edodes*, *L. aquarrosulus*, *Lentinus* TAFRS007, *Lentinus* TAFRS011, *Lentinus* TAFRS014, *M. esculenta*, *P. eryngii*, *P. sajor-caju*, *P. igniarius*, *T. fuciformis* and *V. volvacea*, are commercially available in Thailand as sources of food and nutraceuticals. People consume them in the forms of fresh and dried fruiting bodies in food as well as mycelial powder in food supplements. Therefore, fresh and dried fruiting bodies and mycelia were used in this study. As different solvents can yield different kinds of substances and bioactivities, 3 different solvents with varied polarities were used (Faccin et al., 2007, Sun et al., 2011). The mushroom powder obtained from the extraction process was prepared in 100% DMSO to a final concentration of 100 mg/ml before use.

### 3.2 HIV-1 reverse transcriptase relative inhibition

**Fig. 2:**
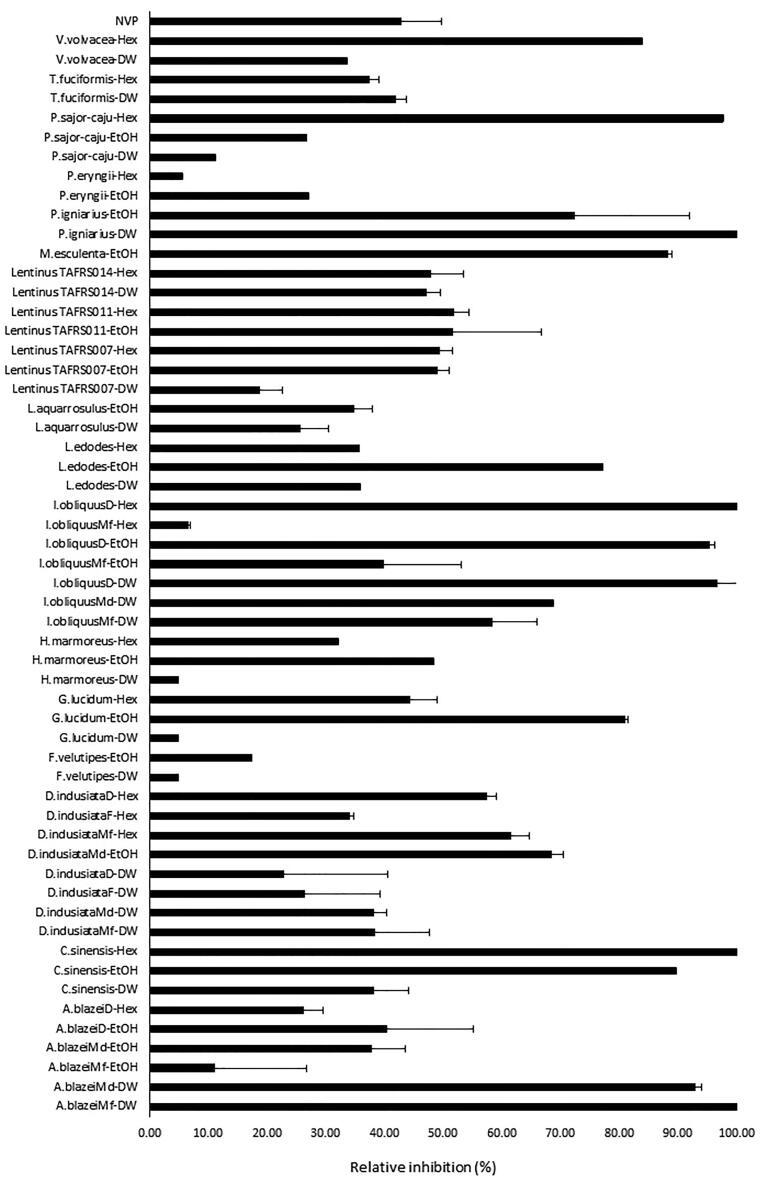
Percent relative inhibition of 0.8 µM NVP and 0.8 mg/ml of each mushroom crude extract against HIV-1 RT.

The relative inhibition of HIV-1 RT by mushroom crude extracts was performed by incubating 0.8 mg/ml of each mushroom with 10 nM purified recombinant wild-type HIV-1 RT. The percent relative inhibition was calculated following methods by Silprasit et al. (2011). The results revealed that mushroom crude extracts of *A. subrufescens*D-DW, *A. subrufescens*Md-Hex, *D. indusiata*Mf-EtOH, *D. indusiata*F-EtOH, *D. indusiata*D-EtOH, *D. indusiata*Md-Hex, *F. velutipes*-Hex, *H. erinaceus*-DW, *I. obliquus*Md-EtOH, *I. obliquus*Md-Hex, *Lentinus* TAFRS011-DW, *Lentinus* TAFRS014-EtOH, *M. esculenta*-DW, *M. esculenta*-Hex, *P. igniarius*-Hex, *P. eryngii*-DW, *T. fuciformis*-EtOH and *V. volvacea*-EtOH showed no inhibition activity against HIV-1 RT. Other mushroom crude extracts showed various percent inhibition activities as shown in Fig. 2. The crude extracts of *A. subrufescens*Mf-DW, *A. subrufescens*Md-DW, *C. militaris*-EtOH, *C. militaris*-Hex, *G. lingzhi*-EtOH, *I. obliquus*D-DW, *I. obliquus*D-EtOH, *I. obliquus*D-Hex, *L. edodes*-EtOH, *M. esculenta*-EtOH, *P. sajor-caju*-Hex, *P. igniarius*-DW, *P. igniarius*-EtOH and *V. volvacea*-Hex showed > 70% percent relative inhibition, thus they were selected for the IC_50_ assay.

### 3.3 Determination of IC_50_

**Fig. 3:**
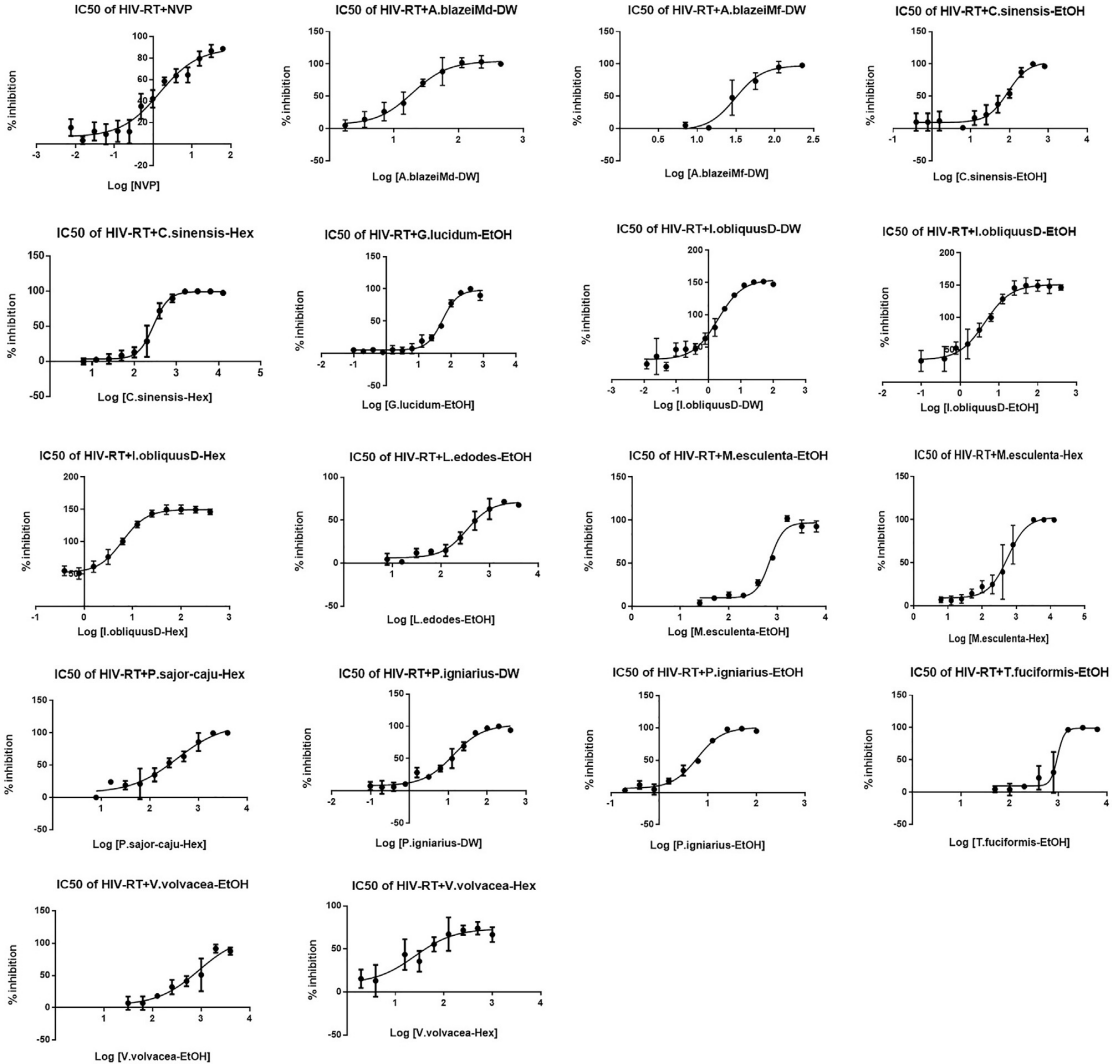
Non-linear regression dose-response plot determining the IC_50_ values of NVP and the selected mushroom crude extracts.

The determination of the IC_50_ of mushroom crude extracts against HIV-1 RT was performed by using the final concentrations of the extracts ranging from 0.012 µg/ml to 32 mg/ml. The nonlinear regression dose–response curves of NVP and the selected mushroom extracts were generated to calculate the IC_50_ values (Fig. 3), and the IC_50_ values are summarized in Table 2. NVP showed the IC_50_ value of 0.39 ± 0.10 µg/ml. The extracts of *I. obliquus*D-DW, *I. obliquus*D-EtOH, *P. igniarius*-EtOH, *I. obliquus*D-Hex and *A. subrufescens*Mf-DW showed strong anti-HIV-1 RT activity with IC_50_ values of 1.92 ± 0.15, 4.39 ± 0.79, 6.05 ± 0.66, 6.17 ± 0.76 and 7.75 ± 2.46 µg/ml, respectively. Other selected mushroom crude extracts showed moderate and weak inhibition activity with IC_50_ values ranging from 13.30 ± 4.10 µg/ml to 0.96 ± 0.41 mg/ml (Table 2).

**Table 2:** The IC_50_ values of NVP and the selected mushroom crude extracts against HIV-1 RT. IC_50_ values lower than 10, 100 and 1,000 µg/ml were considered as strong, medium and low inhibitions, respectively, while a value higher than 1,000 µg/ml was considered as non-inhibition.

**Sample code**	**IC_50_ (µg/ml)**
NVP	0.39 ± 0.10
*A. subrufescens*Mf-DW	7.75 ± 2.46
*A. subrufescens*Md-DW	19.24 ± 9.00
*C. militaris-*EtOH	90.07 ± 8.47
*C. militaris*-Hex	287.10 ± 75.68
*G. lingzhi*-EtOH	54.38 ± 2.29
*I. obliquus*D-DW	1.92 ± 0.15
*I. obliquus*D-EtOH	4.39 ± 0.79
*I. obliquus*D-Hex	6.17 ± 0.76
*L. edodes*-EtOH	342.10 ± 87.26
*M. esculenta*-EtOH	705.50 ± 24.88
*M. esculenta*-Hex	558.80 ± 328.88
*P. sajor-caju*-Hex	364.40 ± 31.54
*P. igniarius*-DW	13.30 ± 4.10
*P. igniarius-*EtOH	6.05 ± 0.66
*T. fuciformis*-EtOH	964.20 ± 408.14
*V. volvacea*-EtOH	886.40 ± 735.04
*V. volvacea*-Hex	25.21 ± 9.34
*P. igniarius*-Hex	2,723.00 ± 690.05

Values were expressed as mean ± SD (n = 3).

## 4 Discussion

The 19 mushrooms, which were *A. subrufescens*, *C. militaris*, *D. indusiata*, *F. velutipes*, *G. lingzhi*, *H. erinaceus*, *H. marmoreus*, *I. obliquus*, *L. edodes*, *L. aquarrosulus*, *Lentinus* TAFRS007, *Lentinus* TAFRS011, *Lentinus* TAFRS014, *M. esculenta*, *P. eryngii*, *P. sajor-caju*, *P. igniarius*, *T. fuciformis* and *V. volvacea*, are commercially available in Thailand as sources of food and nutraceuticals. Several researches have reported that these mushrooms showed various medical properties such as antimicrobial, antiviral, antioxidant, antitumor, immunomodulatory and antiatherosclerotic properties (Lindequist et al., 2005, Mori et al., 2008, Ma et al., 2012). However, studies of antiviral activity of mushroom extracts against HIV-1 have been limited in number. Therefore, these mushrooms were chosen to test whetherthey showed any therapeutic values, especially anti-HIV-1 RT activity. The results revealed that extracts of these mushrooms showed varied anti-HIV-1 RT activity. IC_50_ values lower than 10, 100 and 1,000 µg/ml were considered strong, medium and low inhibitions, respectively, while alues higher than 1,000 µg/ml were considered as non-inhibitions.

The mushrooms used in this study are characterized into the fungal orders *Agaricales*, *Hypocreales*, *Hymenochaelales, Pezizales* and *Polyporales* which have been known to be great sources of nutrients and therapeutic agents (Rahi and Malik, 2016, Linnakoski et al., 2018). Several studies have reported that the anti-viral agents from these fungal orders were polysaccharides, proteins, terpenes as well as phenolic compounds (Faccin et al., 2007, Sun et al., 2011, Dasgupta and Acharya, 2019).

Polysaccharides are major compounds in mushrooms. The most prevalent polysaccharide is β-glucan, which makes up approximately 8–55% of mushroom dry weight (McCleary and Draga, 2016). The isolated polysaccharides from *A. blazei* possessed antiviral activity against herpes simplex type 1 (HSV-1), HSV-2, bovine herpes type 1 (BoHV-1) and poliovirus. An extract of *A. blazei* at a concentration of 100 µg/ml inhibited HSV-1 infection by 78.4% (Bruggemann et al., 2006). The isolated polysaccharide exhibited an IC_50_ value of 97.2 µg/ml against poliovirus by plaque assay (Faccin et al., 2007). The aqueous extracts from stroma and sclerotia of *C. sinensis* inhibited HIV-1 RT (Zhu et al., 2016); unfortunately, the structure of this bioactive compound has not yet been isolated and characterized. The acidic polysaccharide from its related species, *C. militaris*, showed anti-influenza A virus activity (Ohta et al., 2007). The acidic protein bound polysaccharide of *G. lucidum* exhibited EC_50_ values of 300 and 440 µg/ml against HSV-1 and HSV-2, respectively (Eo et al., 2000). The sulfated lentinan, a β-1,3-glucan with a β-1,6-branching polysaccharide from *L. edodes*, showed IC_50_ against poliovirus and bovine herpes virus ranging from 0.19 to 12.7 mg/ml as monitored by plaque assay (Rincão et al., 2012). Polysaccharides might play roles in modulating the immune function of macrophages, interfering with the attachment and absorption of virus to host cells, inhibiting cell-to-cell spread, reducing expression of viral protein or, in this case, inhibiting viral enzyme activity (Bruggemann et al., 2006, Faccin et al., 2007, Ohta et al., 2007, Linnakoski et al., 2018).

Many proteins such as laccases, lectins, anti-fungal proteins, ribosome-inactivating proteins (RIPs) and ubiquitin-like proteins exhibitanti-viral properties. They can be classified as high-molecular weight bioactive compounds, and are water soluble. They showed varied IC_50_ values against HIV-1 RT. Laccases from *G. lucidum* and *L. edodes* inhibited anti-HIV-1 RT with IC_50_ values of 0.12 and 0.50 g/l, respectively (Sun et al., 2011, Wang and Ng, 2006). Laccases were also found in *A. blazei* (Ullrich et al., 2005), *M. esculenta* (Dayi et al., 2018) and *P. sajor-caju* (Khan et al., 2016); however, their anti-HIV-1 RT activities have not been reported yet. Many laccases (such as from *G. lucidum* and *L. edodes*) possess anti-HIV-1 RT activity; it might be postulated that laccases in the extracts of *A. blazei*, *M. esculanta*, *P. sajor-caju* and *V. volvacea* might be responsible for anti-HIV-1 RT properties in this study. Lectins isolated from *H. erinaceum* exhibited anti-HIV-1 RT activity with an IC_50_ value of 1.62 g/l (Li et al., 2010). Lectins have been isolated from *A. blazei* (Kawagishi et al., 1988) and *G. lucidum* (Kawagishi et al., 1997) but their anti-HIV-RT activities have not yet been studied. Lentin, an anti-fungal protein from *L. edodes*, inhibited HIV-1 RT with an IC_50_ of 0.04 g/l (Ngai and Ng, 2003). RIPs from *F. velutipes* (velutin) and *H. marmoreus* (marmorin) exhibited anti-HIV-1 RT activities, and they showed IC_50_ values of 0.41 and 0.29 g/l, respectively (Wong et al., 2008). Ubiquitin-like proteins with anti-HIV-1 RT properties have been isolated from *Agrocybe cylindracea* and *P. ostreatus* (Guillamon et al., 2011), but their inhibition mechanisms await elucidation. The inhibition mechanism of HIV-1 RT by these proteins is yet to be fully resolved but is probably due to protein–protein interaction (Bottcher and Grosse, 1997). Thus, further studies will be required to clarify whether these proteins are responsible for anti-HIV-1 RT activity or not, especially in the distilled water extracts of *A. subrufescens*, *I. obliquus* and *P. igniarius*.

Mushroom terpenes also show antiviral properties. A number of terpenes have been isolated from both medicinal and edible mushrooms, including *G. lucidum*, *I. obliquus*, *P. igniarius*, *F. veltipes*, *H. erinaceus* and *P. eryngii* (Duru and Cayan, 2015, Zapora et al., 2016, Dasgupta and Acharya, 2019). To date, only terpenes from the mushroom genus *Ganoderma* have been investigated for anti-HIV properties. Thirteen triterpene compounds isolated from methanol extracts of the fruiting bodies of *G. lucidum*. Ganoderiol F and ganodermanontriol at a concentration of 7.8 µg/ml completely inhibited HIV-1 induced cytopathic effects in MT-4 cells. Ganoderic acid B inhibited HIV-1 protease with an IC_50_ value of 0.088 g/l (El-Mekkawy et al., 1998). An alcohol solvent (methanol) was used to extract triterpenes from *G. lucidum* The alcohol solvent used in this study (ethanol) could also extract terpenes from *C. militaris*, *I. obliquus*, *L. edodes*, *M. esculenta* and *P. igniarius*. Further study will be required to isolate and identify specific terpenes from these mushrooms, and an inhibition study against HIV-1 RT will be performed.

Plants are known to contain polyphenols with anti-HIV-1 activity (Andrae-Marobela et al., 2013). Polyphenols in mushrooms also exhibited the similar property (Lee and Yun, 2011; Wang et al., 2014). Plant gallic acid derivatives 1,2,6-trigalloylglucopyranose and 1,2,3,6-tertagalloylglucopyranose, which were isolated from stem-bark of *Juglans mandshurica*, exhibited a strong anti-HIV-1 RT property, with IC_50_ values of 42.65 and 30.94 ng/ml, respectively (Min et al., 2002). The mushroom polyphenol methyl gallate, isolated from water and ethanol extracts of *Pholiota adiposa*, inhibited HIV-1 replication in TZM-BL cells infected by a pseudovirus as well as HIV-1 RT and integrase with IC_50_ values of 2.19, 14.75 and 42.07 µg/ml, respectively (Wang et al., 2014). Other compounds such as hispidin and hispolon extracted from *I. hispidus* showed anti-influenza A virus (H1N1 and H3N2) and B (Awadh et al., 2003). Hispidin is also found in *I. obliquus* and *P. igniarius* (Lee and Yun, 2011). The water and ethanol extracts of *I. obliquus* and *P. igniarius*, which showed strong HIV-1 RT inhibition, might contain hispidin. Again, further experiments will be carried out to clarify whether hispidin or other bioactive componds is responsible for the anti-HIV-1 RT activity in these extracts.

It is noteworthy that the use of solvents to extract anti-HIV-1 RT compounds from mushrooms should be carefully considered. A study by El-Mekkawy et al. (1998) showed that a methanol extract of *G. lucidum* showed no anti-HIV-1 RT activity, whereas its acetone extract from a study of Mizushina et al. (1998) showed activity against HIV-1 RT. In addition, the ethanol extract of the mushrooms in this study exhibited good anti-HIV-RT. This phenomenon was also observed in the results comparison between the study by Mlinaric et al. (2005) and this study. The methanol extracts of *F. velutipes*, *G. lucidum* and *L. edodes* showed no anti-HIV-1 RT properties while their ethanol extracts exhibited varied anti-HIV-1 RT potentials. Isolation of the compounds from methanol and acetone extracts revealed that terpenes and cerebrosides were the bioactive substances in these extracts, respectively. The solvent used for extraction of mushrooms might affect the substances obtained. Therefore, the solvent used for extraction of anti-HIV-1 RT substances from mushrooms should take this into account.

Among the 19 mushrooms tested in this study, 4 mushrooms were found to be potential sources of anti-HIV-1 RT substances. Three different extraction solvents were used, and they might yield different substances against HIV-1 RT (Table 3). Suitable isolation protocols to obtain these substances will be used.

**Table 3:** Possible bioactive substances against HIV-1 RT in mushroom extracts.

**Mushrooms**	**Solvents used**	**Possible bioactive substances**	**References**
*A. subrufescens*	Distilled water	β-glucan	Bruggermann et al. (2006)
		Laccase	Ullrich et al. (2005)
		Lectin	Kawagishi et al. (1988)
*I. obliquus*	Distilled water	Terpenes	Pavlova et al. (2003)
			Gery et al. (2018)
	Ethanol	Polysaccharides	Ma et al. (2012)
		Terpenes	Duru et al. (2019)
		Polyphenols	Lee and Yun (2011)
	Hexane	Terpenes	Lindequist et al. (2005)
*P. igniarius*	Ethanol	Terpenes	Zapora et al. (2016)
		Polyphenols	Lee and Yun (2011)

Mushroom extracts have been applied to HIV-infected patients. Application of β-glucan extracted from *Grifola frondosa* showed a positive impact in HIV-infected patients. It increased CD4^+^ cell count and sense of well-being (Nanba et al., 2000). An increase in CD4^+^ cell count was also observed in a small-scale study in Ghana by Adotey et al. (2011). Moreover, mushroom extracts with anti-fungal activity such as *G. lucidum* and *Termitomyces titanicus* could be used in the treatment of opportunistic skin infectious diseases in HIV-infected patients. Both mushrooms showed 95% efficacy to treat skin diseases caused by *Tinea capitis*, *T. corporis*, *T. pedis* and *T. unguium* (Yongabi et al., 2014). Besides using crude extracts in inhibition of HIV-1, combination usage of these compounds with anti-HIV drugs are applicable. Combined use of sulfated lentinan from *L. edodes* and 3′-azido-3′-deoxythymidine (AZT) showed more efficient suppression of in vitro expression of HIV antigens when compared to the use of AZT alone (Tochikura et al., 1987). A phase II study conducted by Gordon et al. (1995) revealed that the combination treatment of lentinan and the HIV-1 RT inhibitor didanosine resulted in a significant increase in the number of CD4^+^ cells when compared to didanosine monotherapy. Lentinan is known to possess immunostimulating activity, and it might help anti-HIV-1 drugs by blocking HIV infection or interfering with HIV replication through production of various factors such as cytokines (Zhang et al., 2011). HIV infection and highly active antiretroviral therapy (HAART) can cause oxidative stress. Nutrition supplementation with mushroom extracts could reduce oxidative stress in HIV patients (Figueira et al., 2014). From these findings, mushroom extracts can be used to treat HIV-infected patients in the form of crude extracts/purified bioactive substances or sole extracts/drug tonics against HIV itself as well as be a part of nutrition supplementation or skin cream to treat skin infection.

## 5 Conclusions

The distilled water, ethanol and hexane crude extracts of 19 edible and medicinal mushrooms were screened against HIV-1 RT. The results revealed that water extracts of *A. subrufescens* and *I. obliquus*, ethanol extracts of *I. obliquus* and *P. igniarius* and a hexane extract of *I. obliquus* exhibited strong anti-HIV-1 RT activity with IC_50_ values of 1.92–7.75 µg/ml. Among the 19 mushrooms tested, 4 mushrooms which were *A. subrufescens*, *I. obliquus*, *P. igniarius* and *V. volvacea* were firstly reported in this study to have anti-HIV-RT properties. The bioactive substances responsible for anti-HIV-1 RT activity will be identified and characterized. Polysaccharides or some fungal proteins such as laccase, lectin and RIPs will be extracted from distilled water extracts, whereas terpenes and polyphenols will be extracted from ethanol and hexane extracts of mushrooms. In addition, further study is also required to investigate whether the anti-HIV-1 RT is due to a specific component or combined effect of various individual constituents.

## Declaration of Competing Interest

The authors declare that they have no known competing financial interests or personal relationships that could have appeared to influence the work reported in this paper.
